# Community-acquired Oseltamivir-Resistant Pandemic (H1N1) 2009 in Child, Israel

**DOI:** 10.3201/eid1606.091875

**Published:** 2010-06

**Authors:** Zeev Zonis, Dan Engelhard, Musa Hindiyeh, Daniela Ram, Michal Mandelboim, Ella Mendelson, Daniel Glikman

**Affiliations:** Western Galilee Hospital, Nahariya, Israel (Z. Zonis, D. Glikman); Hadassah University Hospital, Jerusalem, Israel (D. Engelhard); Israel Ministry of Health, Public Health Services and Chaim Sheba Medical Center, Tel-Hashomer, Israel (M. Hindiyeh, D. Ram, M. Mandelboim, E. Mendelson)

**Keywords:** Pandemic (H1N1) 2009, oseltamivir resistance, acute respiratory distress syndrome, ARDS, child, Israel, influenza, viruses, letter, expedited, Suggested citation for this article: Zonis Z, Englehard D, Hindiyeh M, Ram D, Mandelboim M, Mendelson E, et al. Community-acquired oseltamivir-resistant pandemic (H1N1) 2009 in child, Israel [letter]. Emerg Infect Dis [serial on the Internet]. 2010 Jun [*date cited*]. http://www.cdc.gov/EID/content/16/6/1045.htm

**To the Editor:** During the spring of 2009, a pandemic influenza A (H1N1) virus emerged and spread globally. Initial testing of the virus found it susceptible to neuraminidase inhibitors and resistant to adamantanes (*1*,*2*). As of March 5, 2010, only 264 cases of oseltamivir-resistant pandemic (H1N1) 2009 infection had been reported to the World Health Organization, but the number of cases has been steadily increasing (*2*). These viruses were carrying the H275Y mutation, which conferred resistance to oseltamivir (*2*). Most of the reported cases were in immunocompromised patients who had prolonged viral shedding or in patients who had received oseltamivir prophylaxis or treatment (*1*–*4*). We describe an otherwise healthy 2-year-old boy with oseltamivir-resistant pandemic (H1N1) 2009 infection and a traumatic lung contusion, complicated by acute respiratory distress syndrome (ARDS). He had not received prior chemoprophylaxis or treatment with oseltamivir.

In November 2009, a healthy 2-year-old boy was admitted to the pediatric intensive care unit at the Western Galilee Hospital in Nahariya, Israel, after he had been hit by a car. One day before the accident, he had exhibited fever and cough (for which he was treated with acetaminophen). His 4-year-old brother had recovered recently from an influenza-like illness without antiviral treatment. The other household contacts were his parents, who did not have a respiratory illness.

On admission, small, bilateral lung contusions, right pneumothorax, and liver lacerations were shown on computed tomographic scan. The patient was treated with a chest tube for drainage, supplemental oxygen, and oseltamivir from hospital day 1 (30 mg 2 ×/day; child's body weight = 13 kg) and was placed in droplet isolation. Respiratory swab specimens, obtained on hospital day 1, were sent to the Israel Central Virology Laboratory (ICVL) and found to be positive for pandemic (H1N1) 2009 by real-time reverse transcription–PCR (RT-PCR). On hospital day 3, the child was intubated because of worsening respiratory distress and hypoxemia, and he required a second chest tube drain. His chest film showed bilateral pulmonary infiltrates. His condition was then treated with nitric oxide, dopamine, and milrinone for ARDS and failure of the right side of the heart. The dosage of oseltamivir was doubled on hospital day 4 because of gastric residuals. Antimicrobial drug therapy with vancomycin and piperacillin-tazobactam was added because sepsis and secondary bacterial lung infection were suspected. Because of the severity of his symptoms and persistence of fever, additional lower and upper airway specimens were sent to ICVL on hospital days 5 and 10; they were positive for pandemic (H1N1) 2009.

After these results were received, oseltamivir resistance was suspected, and his respiratory specimens were also checked by ICVL. A mixture of both wild-type and mutant pandemic (H1N1) 2009 was found in the specimens from hospital days 1, 5, and 10 by an in-house q-RT-PCR assay designed to detect the H275Y mutation (*4*,*5*). Further testing by sequence analysis of the neuraminidase gene showed a mixed population of wild-type and mutant pandemic (H1N1) 2009; the mutant virus was carrying the histidine-to-tyrosine substitution at position 275, which conferred the quantitative RT-PCR result and the H275Y phenotype of oseltamivir-resistant pandemic (H1N1) 2009. By the time these laboratory results were known, the patient’s respiratory condition was improving without changing the oseltamivir therapy. Cultures of blood and endotracheal specimens were sterile and antimicrobial drug therapy was stopped. On hospital day 15, he was extubated, oseltamivir therapy was ended, and he was weaned off oxygen a few days later. The respiratory specimen on hospital day 20 was negative for pandemic (H1N1) 2009. No secondary influenza cases were detected among healthcare personnel or patients in the unit.

In Israel, oseltamivir resistance has been detected by ICVL in 6 cases (*5*). The fact that our patient had oseltamivir-resistant pandemic (H1N1) 2009 without a previous oseltamivir exposure is surprising because almost all cases of oseltamivir-resistance have been associated with previous oseltamivir prophylaxis or therapy and with prolonged viral shedding (which is often combined with oseltamivir therapy) in immunocompromised patients (*1*–*5*). Our patient did not attend daycare and his parents had not been ill recently. Therefore, he likely was infected by his older brother who probably had pandemic (H1N1) 2009 but was neither diagnosed nor treated with antiviral medications. This theory suggests that oseltamivir-resistant viruses circulate in the community with the potential to be transmitted between persons.

Lung contusions and pandemic (H1N1) 2009 can cause ARDS (*6*,*7*). We do not know the relative role of each in causing the ARDS that our patient had, but the severity of clinical symptoms, although the lung injury was judged to be only of moderate magnitude, suggests that influenza played a major role in the development of his acute lung disease. The infection with oseltamivir-resistant virus, for which he probably did not receive effective therapy, likely had an effect on the duration and severity of his course.

Although our patient had a favorable outcome, the possibility of widespread resistance, similar to the phenomenon observed with seasonal influenza in the 2008–2009 season, is alarming and should be monitored. The suspicion of resistance should be based upon compatible clinical scenario, such as continuation of symptoms in spite of antiviral therapy (even in patients who are not immunocompromised), combined with early performance of resistance assays. Early and rapid detection of oseltamivir resistance and a change of antiviral treatment (if feasible) might benefit the patient.
